# Daily Social Life of Older Adults and Vulnerabilities During the COVID-19 Pandemic

**DOI:** 10.3389/fpubh.2021.637008

**Published:** 2021-08-17

**Authors:** Jen-Hao Chen

**Affiliations:** Department of Sociology and Department of Psychology, National Chengchi University, Taipei, Taiwan

**Keywords:** age and gender differences, daily life, time use, social contact, social inequality

## Abstract

**Introduction:** The social integration of older adults is crucial for understanding their risk of infection and mental health during the COVID-19 pandemic. However, the social lives of older adults differ, which means they are not all vulnerable to COVID-19 in the same way. This study analyzes everyday time use and social contacts of older adults to inform discussions of their vulnerabilities during the pandemic.

**Methods:** Using the 2019 American time use survey (*N* = 4,256, aged 55 and older), hurdle model regressions were used to examine the relationship between age, gender, and six indicators of the degree of social contact and time use, including (1) time alone, (2) time spent with family members, (3) time spent with non-family members, (4) time spent with people in the same household, (5) number of public spaces visited, and (6) time spent in public spaces.

**Results:** Results showed substantial heterogeneity in everyday time use and social contacts. Time in public places gradually decreased from the oldest-old (85 years or older), old-old (75–84 years), to mid-life (55–64 years) adults. The gaps were not explained by age differences in sociodemographic characteristics and social roles. Compared with mid-life adults, time with family members of the young-old and old-old adults decreased, but time with non-family members increased. Age differences in social roles over the life course partially explained the differences.

**Conclusions:** Should these patterns of time use and social contacts persist during COVID-19; then, such variations in the organization of social life may create different exposure contexts and vulnerabilities to social distancing measures among older adults; such information could help inform interventions to better protect this population.

## Introduction

The novel coronavirus has significantly disrupted the lives of billions of people. The US was among those hardest hit, with over 33 million confirmed cases and nearly 600,000 deaths by mid-June 2021 (1). Biomedical research findings indicate that older adults have the highest risk of developing serious complications and of dying from COVID-19 (2–4). In addition, reduced material resources and restricted social contact due to the pandemic-induced recession and containment measures have considerably disrupted the lives of older adults and generated tremendous stress and psychological burden. Indeed, a growing literature study has demonstrated the staggering, negative effects of COVID-19 on the mental well-being of older adults worldwide (5–7). As such, protecting older adults from being infected and promoting their mental well-being is of paramount importance.

While we all agree that older adults are vulnerable during the COVID-19 pandemic, the existing discourse on the vulnerabilities of older adults during the COVID-19 pandemic does not take into account the heterogeneity that exists in their social lives (e.g., with whom they have daily social contact and where they visit) and how that relates to their risk of COVID-19 infection and potential psychosocial consequences from social distancing. This is an important omission because coronavirus is a “social virus.” It spreads mainly through person-to-person contact. In addition, social distancing measures that aim to reduce the spread of COVID-19 through maintaining physical distance and reducing social interactions also limit the access of an individual to beneficial social resources and social support (8, 9). As such, the structure of social connectedness not only influences the risk of infection of an individual but also determines their psychosocial consequences in the face of social distancing measures.

Most of the studies that apply a social connectedness perspective on this issue are grounded in the analysis of interpersonal networks. Studies have demonstrated that the social network of an individual is a critical lever that, when changed, can slow down or speed up the spread of the disease [e.g., (10, 11)]. However, the existing studies pay more attention to social network ties, which are typically defined in terms of personal relationships that persist or recur over longer, more indefinite time periods. Relatively little attention has been paid to the extent to which individuals are actually in contact on a daily basis [please also see (12) for a critique of the approach].

It is well-documented that the time doing paid work reduces and the time doing leisure activities increase with age (13). Yet, aging research has long recognized that older adults are a heterogeneous group and that the aging process is diverse (14, 15). Weber et al. (16) find that there is minimal difference in terms of diversity of activities and social contact between younger adults and older adults. The variations in the social lives of older adults produce different patterns of everyday social contact and time use, which potentially creates substantial heterogeneity in exposure contexts and moderates the impact of social distancing measures on psychosocial well-being. For example, Cornwell (12), using the 2003–2009 American Time Use Survey (ATUS), finds a decline in daily social contact time with aging. Among older adults, women have spent less time with kin and more time alone than men. Marcum (17) analyzes 2003–2008 ATUS in a different way by focusing on time spent on activities with others. He finds that older age is associated with doing various activities alone. By analyzing daily diaries of two groups of participants from Germany before the pandemic, Weber et al. (16) find that older adults have fewer social contact partners than younger adults, but show greater diversity of daily activities. A research study also suggests that older adults who live alone may spend more time with friends and non-family members (18).

Thus, such diverse patterns of daily life and social contact of older adults can inform the discussion of the risk and consequences during the pandemic. For example, the risk of COVID-19 infection in public places is different for an older adult who lives alone and spends a lot of time outside than it is for an older adult who mostly stays at home and interacts primarily with family members. The former has a higher risk of infection from non-family members, whereas the latter has a higher risk of infection at home and from family members. In other words, while every senior is considered high risk, they are not all vulnerable to COVID-19 in the same way. Understanding different groups of older adults' daily social contact and time use will help governments develop better strategies to prevent COVID-19 transmission and promote psychosocial well-being during the stay-at-home period in this heterogeneous, old-age population.

Given the importance of the extent to which older adults are actually in contact on a daily basis, this study proposes rethinking the risk of COVID-19 infection and vulnerabilities of older adults under social distancing measures through a careful examination of their everyday social contacts and time use. Understanding the heterogeneity in daily social contacts and time use of older adults can shed light on where and to whom older adults are exposed, which context each group of older adults is most or least vulnerable to, and in turn, what preventive measures would be most effective. It is important to note that this analysis of social contact and time use is a distinct approach that differs from the social network approach [e.g., (19–21)] that has informed the discourse. First, consider that everyday person-to-person contact occurs in a wide range of settings, such as in a library, mall, or restaurant, and is not limited to members of the social network of a person. Since this new coronavirus is so highly contagious in face-to-face interactions, focusing only on the interpersonal social networks of older adults misses important information about social contacts that affects their risk of infection. Second, consider that having close network members and being socially active does not necessarily translate to intensive and frequent social contact in daily life. For example, available telecommunication technology means an older adult can maintain emotional closeness to their network members and receive tremendous social support, without frequent person-to-person contact. By placing daily social contact at the center of the analysis, instead of interpersonal social networks or levels of social engagement, this study is able to better assess the heterogeneity in the exposure contexts of older adults for COVID-19 infection.

## Methods

### Data: 2019 ATUS

This study used data from the most recent wave (2019) of the American Time Use Survey (ATUS). The ATUS is a national representative survey that collects 24-h time diaries of Americans aged 15 years and older. Each respondent is asked to provide detailed information on activities for one randomly selected day, starting at 4:00 a.m. the previous day and ending at 4:00 a.m. on the day of the interview. The information collected includes the duration of each activity, where the activity took place, and who the activity was with. This is where time use data comes alive and becomes valuable: These data allow for assessing the extent and degree of social contact in everyday life by people of different age groups. Detailed information on the ATUS can be found elsewhere (22). The 2019 ATUS had 9,435 respondents. This study limited respondents to persons aged 55 years and over (*N* = 4,256). To further assess the differences in everyday social contact within the population of midlife and older adults, this study followed the tradition in the gerontology literature and further distinguishes the midlife and older population into four age groups, namely, mid-life (55–64 years), young-old (65–74 years), old-old (75–84 years), and oldest-old (85 years or older).

### Measures of Everyday Social Contact

Using detailed information about the time use of the respondents, this study used time diaries to create six indicators that reflect the degree of social contact of a respondent. They are as follows: (1) time alone, defined as the total amount of non-sleeping time alone, regardless of the activity; (2) time with immediate family members, defined as the total amount of non-sleeping time with spouses/partners, parents, children, grandchildren, and siblings; (3) time with non-family members, defined as the total amount of non-sleeping time with people who are not immediate family members; (4) time with people in the same household, defined as the total amount of non-sleeping time with people who live in the same household with the respondent; (5) time spent in public places, defined as the total amount of time the respondent stayed in a workplace, restaurant, place of worship, grocery store, mall, library, gym, post office, and public transportation; and (6) total number of public places visited during a day. To create the first five measures, this study summed the total minutes from a 24-h time-diary data that each respondent spent their time. For example, to create a measure of time alone, this study summed the time alone of each respondent, regardless of the activities. Time with immediate family members, time with non-family members, time with people in the same household, and time in a public place are calculated using the same method. It is worth noting that these measures might not be mutually exclusive. For example, if a respondent spent 30 min in a library alone, the time would be counted toward both indicators of time alone and time in public places. For the last measure (total number of public places visited during a day), this study counts the number of non-home places that each respondent visited during the selected day.

### Covariates

This study also took advantage of the rich sociodemographic information of ATUS data and controlled for sociodemographic characteristics and functional limitations of the respondents because these factors are also predictors of the social contact and time use of an individual (12, 17, 23). These included gender, race, and ethnicity (White, Black, Hispanic, and others), education (less than high school, high school, some college, and college or above), and an indicator of weekend time-diary. In ATUS, respondents were asked to report their total yearly family income in categories. A total of 16 income categories are provided, ranging from <5,000 dollars per year to over 150,000 dollars per year. This study treated the total family income as a continuous variable in the following statistical analysis because treating it as a categorical variable yields the same findings. This study also controlled for functional limitations. The ATUS includes a series of six questions that assess the disabilities of respondents. This study created a scale that summed the number of disabilities of the respondents (23, 24). Finally, this study also included the marital status (married, widowed, divorced, and never married) and living arrangements (with spouse only, living alone, intergenerational household, and other arrangements) of the respondents.

### Statistical Analysis

This study used the double-hurdle model to link age group to the degree of social contact. The hurdle model is used for zero-inflated data and can be applied to the analysis of time use data (25). The application of the hurdle model involved two steps. The first step specified the process that affected the likelihood of having a specific type of social contact (i.e., whether the respondent engaging in the specific kind of everyday social contact) of the respondents. The second step specified the process that influenced the duration of a specific type of social contact of the respondents.

Two models were conducted. Model 1 controlled for gender, race and ethnicity, family income, functional limitations, and whether it is a weekend time-diary. Model 2 included covariates that captured the social roles of an individual by adding marital status, living arrangements, and employment status. By comparing results from Model 1 and Model 2, it allowed for an assessment of the effects of changes in social roles in influencing everyday social contact and time use of older adults. This study focused on discussing the results for the duration because they are more relevant to our understanding of heterogeneity in the exposure contexts of older adults. All regressions were weighted using the population weights provided by the ATUS dataset, and so the results can be generalized to the population of older adults in the US. All statistical analyses were conducted using Stata 16.

## Results

Table 1 shows the descriptive statistics of the analytical sample. About 44% of the sample were mid-life adults, followed by 33% young-old adults, 18% old-old adults, and 5% oldest-old adults. The sample had slightly more women (54%) than men. As expected, the majority of the respondents were White, but the analytical sample included 13% Black and 10% Hispanic participants. About 10% of respondents did not have a high school degree. Over half of the sample were currently married. About 44% lived with their spouses only, 14% respondents were widowed, and another 8% lived in an intergenerational household. In addition, a substantial percentage of the respondents (23%) lived in other complex arrangements. Finally, most of the older adults were retired (58%) and only a very small percentage of older adults were considered unemployed (2%).

**Table 1:** Descriptive statistics of selected demographic characteristics the sample (weighted, *N* = 4,256).

	**Mean or proportion**	**Standard deviation**
**Age group**		
Mid-life: 55-64 years	0.44	
Young-old: 65-74 years old	0.33	
Old-old: 75-84 years old	0.18	
Oldest-old:85 or older	0.05	
Female	0.54	
**Race**		
White	0.73	
Black	0.13	
Others	0.04	
Hispanic	0.10	
**Education**		
Less than high school	0.11	
High school	0.35	
Some college	0.21	
College or above	0.33	
Disabilities	0.32	0.84
**Marital status**		
Married	0.60	
Widowed	0.14	
Divorced	0.18	
Never married	0.07	
**Living arrangements**		
With spouse only	0.44	
Living alone	0.25	
Intergenerational household	0.08	
Other arrangements	0.23	
**Employment status**		
Employed	0.41	
Unemployed	0.02	
Retired or not in labor force	0.57	

*This table was based on the analysis of the 2019 American Time Use Survey (ATUS) respondents aged 55 years or older*.

Table 2 shows the degree of social contact in daily life by age group. Results showed that as individuals age, they spent more daily time alone, spent less time with non-family members, visited fewer public places, and stayed in public places for a shorter length of time. Compared with mid-life adults, the total amount of time spent with immediate family members on a daily basis increased among the young-old and old-old but decreased among the oldest-old. These results suggest that the older population is heterogeneous in terms of everyday time use and social contact. Such within-group differences may render different vulnerabilities to COVID-19 infection and consequences of social distancing measures.

**Table 2:** Summary statistics of measures of extent and degree of social contact by age group (weighted, *N* = 4,256).

	**Time alone** **(minutes)**	**Time with immediate family members** **(minutes)**	**Time with non-family members** **(minutes)**	**Time with people in the same household** **(minutes)**	**Time in public places** **(minutes)**	**Number of places visited during a day** **(number)**
**Mid-life: 55-64 years old**						
Mean	434	255	163	197	229	2.8
Standard deviation	283	262	238	227	251	1.8
**Young-old: 65-74 years old**						
Mean (minutes)	455	307	76	241	112	2.6
Standard deviation	300	290	161	262	168	1.8
**Old-old: 75-84 years old**						
Mean (minutes)	469	299	54	237	80	2.3
Standard deviation	301	294	133	279	122	1.9
**Oldest-old: 85 or older**						
Mean (minutes)	557	209	53	159	56	1.8
Standard deviation	311	279	119	258	90	1.7

*This table was based on the analysis of the 2019 (ATUS) respondents aged 55 years or older*.

Table 3 goes deeper by examining the differences in the degree of everyday social contact and time use using the hurdle model regressions. This characterizes the degree of heterogeneity within the older population and assesses the extent to which different social roles contribute to such differences. Model 1 controlled for basic demographic characteristics and disabilities. Model 2 included covariates that captured different social roles in work and family domains. Results from Model 1 showed that compared with mid-life adults, the young-old and the old-old spent more time with family members (Coeff = 74.65, SE = 13.84, *p* < 0.001; Coeff = 71.70, SE = 17.22, *p* < 0.001), spent less time with non-family members (Coeff = −107.7, SE = 16.28, *p* < 0.001; Coeff = −129.1, SE = 19.11, *p* < 0.001), spent more time with people in the same household (Coeff = 82.53, SE = 13.58, *p* < 0.001; Coeff = 100.4, SE = 18.25, *p* < 0.001), and stayed in public places for a shorter length of time (Coeff = −129.3, SE = 11.32, *p* < 0.001; Coeff = −159.6, SE = 12.15, *p* < 0.001). Compared with mid-life adults, the oldest-old spent more time alone (Coeff = 108.9, SE = 26.99, *p* < 0.001), visited public places less frequently (Coeff = −0.39, SE = 0.12, *p* < 0.01), stayed for shorter periods of time (Coeff = −158.0, SE = 16.11, *p* < 0.001), but did not differ in time spent with family members and non-family members.

**Table 3:** Results from hurdle model regressions linking age group to extent and degree of social contact in daily life (*N* = 4,256).

	**Time alone**		**Time with immediate family members**		**Time with non-family members**		**Time with people in the same household**		**Time in public places**		**Number of places visited during a day**	
	**Model 1** **coeff.** **(SE)**	**Model 2** **coeff.** **(SE)**	**Model 1** **coeff.** **(SE)**	**Model 2** **coeff.** **(SE)**	**Model 1** **coeff.** **(SE)**	**Model 2** **coeff.** **(SE)**	**Model 1** **coeff.** **(SE)**	**Model 2** **coeff.** **(SE)**	**Model 1** **coeff.** **(SE)**	**Model 2** **coeff.** **(SE)**	**Model 1** **coeff.** **(SE)**	**Model 2** **coeff.** **(SE)**
**Age group (ref: 55-64 years)**												
Young-old: 65-74 years old	14.98 (12.78)	11.61 (12.63)	74.65^***^ (13.84)	26.85 (13.74)	−107.7^***^ (16.28)	−37.83^*^ (17.23)	82.53^***^ (13.58)	41.71^**^ (13.84)	−129.3^***^ (11.32)	−38.06^***^ (10.67)	−0.04 (0.07)	−0.02 (0.07)
Old-old: 75-84 years old	18.85 (15.39)	−4.62 (15.24)	71.70^***^ (17.22)	22.03 (17.44)	−129.1^***^ (19.11)	−19.97 (21.02)	100.4^***^ (18.25)	54.89^**^ (18.44)	−159.6^***^ (12.15)	−37.54^**^ (11.93)	−0.10 (0.08)	−0.08 (0.09)
Oldest-old:85 or older	108.9^***^ (26.99)	21.91 (25.24)	9.51 (35.00)	5.02 (32.25)	−127.5^***^ (25.18)	−8.62 (29.54)	61.46 (40.84)	38.64 (37.51)	−158^***^ (16.11)	−27.52 (16.32)	−0.39^**^ (0.12)	−0.40^**^ (0.13)
Female	−13.50 (11.33)	−41.78^***^ (10.58)	−4.80 (12.13)	10.32 (11.84)	−36.91^*^ (14.99)	−19.95 (14.66)	−15.06 (11.83)	−12.04 (11.67)	−31.79^**^ (10.35)	−11.60 (9.06)	0.28^***^ (0.06)	0.29^***^ (0.06)
**Race (ref: white)**												
Black	45.86^**^ (14.87)	20.45 (13.75)	−83.44^***^ (17.92)	−59.46^**^ (17.35)	35.93 (21.05)	33.73 (19.05)	−93.74^***^ (17.28)	−78.94^***^ (16.54)	2.18 (15.97)	7.91 (13.31)	−0.15 (0.08)	−0.14 (0.08)
Others	30.78 (30.69)	39.65 (30.39)	−38.92 (41.66)	−44.44 (42.04)	−35.90 (32.26)	−14.03 (31.36)	−19.49 (42.34)	−27.73 (13.44)	−8.80 (27.07)	4.76 (23.47)	−0.19 (0.15)	−0.19 (0.15)
Hispanic	−29.78 (22.98)	−17.64 (20.63)	−2.98 (23.84)	9.13 (21.60)	35.84 (32.12)	25.37 (30.43)	−2.41 (22.94)	5.94 (21.09)	−1.74 (22.86)	−8.22 (19.97)	−0.19 (0.10)	−0.19 (0.10)
**Education (ref: less than high school)**												
High school	35.94 (21.18)	41.50^*^ (19.35)	12.60 (23.68)	−4.05 (21.69)	−50.72 (30.84)	−45.41 (28.36)	9.81 (26.42)	2.28 (25.19)	−25.60 (21.47)	−21.48 (16.83)	−0.05 (0.12)	−0.04 (0.12)
Some college	23.07 (21.32)	21.24 (19.59)	32.49 (24.61)	17.29 (22.74)	−48.45 (30.87)	−49.36 (27.72)	24.22 (27.19)	15.32 (26.09)	−20.23 (21.57)	−19.79 (16.77)	0.00 (0.12)	0.01 (0.12)
College or above	43.89^*^ (21.73)	37.68 (20.19)	6.27 (25.14)	−7.50 (23.31)	−72.76^*^ (31.03)	−69.51^*^ (28.08)	−0.33 (27.39)	−7.44 (26.12)	−48.31^*^ (21.84)	−47.89^**^ (17.11)	0.32^**^ (0.12)	0.33^**^ (0.13)
Family income	−11.14^***^ (1.65)	1.05 (1.55)	−0.75 (1.95)	−1.97 (1.91)	6.05^**^ (2.17)	2.11 (2.02)	−5.62^**^ (2.02)	−3.76 (2.01)	6.07^***^ (1.53)	2.20 (1.26)	0.02 (0.02)	0.02 (0.02)
Disabilities and functional limitations	17.42^*^ (6.95)	3.18 (6.30)	9.63 (7.94)	11.36 (7.41)	−15.72^*^ (7.48)	−3.78 (7.47)	19.11^*^ (8,64)	17.77^*^ (8.53)	−28.19^***^ (6.38)	−8.19 (4.98)	−0.17^***^ (0.04)	−0.16^***^ (0.04)
Weekend time diary	−40.18^***^ (10.10)	−44.74^***^ (9.38)	95.45^***^ (11.13)	101.87^***^ (10.65)	−75.64^***^ (13.59)	−61.64^***^ (13.46)	78/65^***^ (11.44)	80.11^***^ (11.14)	−97.10^***^ (8.50)	−94.44^***^ (7.90)	−0.07 (0.05)	−0.07 (0.05)
**Marital status (ref: married)**												
Widowed		118.72^***^ (25.86)		−129.1^***^ (23.95)		43.21 (36.74)		−138.3^***^ (25.56)		−2.12 (21.33)		−0.10 (0.14)
Divorced		87.43^***^ (24.23)		−53.31^*^ (25.06)		42.13 (32.87)		−59.44^*^ (27.15)		2.54 (21.72)		−0.21 (0.12)
Never married		110.81^***^ (27.48)		−86.52^**^ (30.22)		41.83 (35.64)		−51.35 (37.00)		−5.68 (23.51)		−0.14 (0.14)
**Living arrangements (ref: with spouse only)**												
Living alone		195.74^***^ (25.53)		−114.1^***^ (25.07)		−32.23 (34.72)		NA (NA)		7.80 (22.17)		0.24 (0.13)
Intergenerational household		22.89 (23.65)		−7.97 (24.72)		−39.37 (32.67)		29.37 (21.72)		−24.79 (19.63)		0.19 (0.12)
Other arrangements		60.82^**^ (21.58)		−51.56^**^ (18.24)		−2.91 (29.94)		−19.42 (17.88)		−47.89^**^ (17.11)		0.02 (0.11)
**Employment status (ref: Employed)**												
Unemployed		110.35^*^ (53.70)		46.90 (49.30)		−248.8^***^ (30.59)		70.47 (50.07)		−276.3^***^ (18.92)		−0.15 (0.24)
Retired or not in labor force		27.92^*^ (12.22)		116.18^***^ (12.92)		−198.8^***^ (15.65)		103.69^***^ (12.82)		−233.6^***^ (9.69)		−0.06 (0.07)

* *P < 0.05*,

** *P < 0.01, and*

*** *P < 0.001. This table was based on the analysis of the 2019 (ATUS) respondents aged 55 or older. All regressions were weighted*.

Results from Model 2 showed that, after accounting for social roles in work and family domains, some of the gaps in everyday time use and social contact disappeared. For example, after accounting for marital status and living arrangements, there was no statistical difference in time alone across age groups. This suggests that oldest-old adults are more likely to spend time alone than mid-life adults because they are more likely to live alone and be widowed. Among young-old and old-old adults, they still spent less time with non-family members (Coeff = −37.83, SE = 17.23, *p* < 0.05), stayed in public places for a shorter duration (Coeff = −38.06, SE = 10.67, *p* < 0.001; Coeff = −37.54, SE = 11.93, *p* < 0.01), and spent more time with people in the same household (Coeff = 41.71, SE = 13.84, *p* < 0.01; Coeff = 54.89, SE = 18.44, *p* < 0.01). Yet, the coefficients were substantially reduced after accounting for marital status, living arrangements, and employment status. Finally, oldest-old adults still visited fewer public places than mid-life adults (Coeff = −0.40, SE = 0.13, *p* < 0.01), and the association was not explained by differences in social roles.

Table 4 shows age differences in everyday social contact and time use by gender. Results from Model 1 showed some gender differences in everyday time use. For example, compared with men, old-old women and oldest-old women spent more time alone (Coeff = 84.94, SE = 31.17, *p* < 0.01; Coeff = 129.8, SE = 55.19, *p* < 0.05) and stayed longer in public places (Coeff = 48.82, SE = 24.16, *p* < 0.05; Coeff = 80.16, SE = 30.48, *p* < 0.01). Compared with the oldest-old men, the oldest-old women also spent substantially less time with family members (Coeff = −137.3, SE = 64.80, *p* < 0.05). Moving to Model 2, results showed that most of the gender differences in everyday time use and social contact could be accounted for by gender differences in marital status, living arrangements, and employment status. After these variables were included, most of the interaction terms were statistically insignificant.

**Table 4:** Results from hurdle model regressions linking age group to extent and degree of social contact in daily life, with gender interactions (*N* = 4,256).

	**Time alone**		**Time with immediate family members**		**Time with non-family members**		**Time with people in the same household**		**Time in public places**		**Number of places visited during a day**	
	**Model 1** **coeff.** **(SE)**	**Model 2** **coeff.** **(SE)**	**Model 1** **coeff.** **(SE)**	**Model 2** **coeff.** **(SE)**	**Model 1** **coeff.** **(SE)**	**Model 2** **coeff.** **(SE)**	**Model 1** **coeff.** **(SE)**	**Model 2** **coeff.** **(SE)**	**Model 1** **coeff.** **(SE)**	**Model 2** **coeff.** **(SE)**	**Model 1** **coeff.** **(SE)**	**Model 2** **coeff.** **(SE)**
**Age group (ref: 55-64 years)**												
Young-old: 65-74 years old	6.18 (20.19)	15.76 (19.25)	65.18^**^ (20.90)	4.55 (20.08)	−93.01^***^ (26.39)	−29.21 (30.68)	60.98^**^ (19.24)	14.45 (19.59)	−137.2^***^ (17.82)	−45.54^**^ (16.29)	−0.10 (0.09)	−0.06 (0.09)
Old-old: 75-84 years old	−27.57 (24.64)	−11.83 (23.04)	86.98^**^ (26.26)	5.45 (25.28)	−118.5^***^ (31.4)	−39.06 (35.35)	78.99^**^ (25.04)	18.41 (24.79)	−185.5^***^ (18.59)	−51.68^**^ (17.07)	−0.04 (0.11)	0.02 (0.12)
Oldest-old:85 or older	27.36 (45.73)	−5.36 (38.99)	89.47^*^ (43.38)	16.15 (39.69)	−183.4^***^ (38.8)	56.47 (48.28)	123.8^**^ (43.83)	59.12 (43.80)	−202.0^***^ (21.68)	−52.88^*^ (22.14)	−0.19 (0.16)	−0.18 (0.17)
Female	−39.73^*^ (17.67)	−43.20^*^ (17.13)	−0.50 (17.59)	−7.99 (16.66)	−29.56 (22.82)	−8.89 (21.50)	−31.99^*^ (15.90)	−39.66^*^ (15.44)	−47.15^**^ (17.91)	−21.74 (15.32)	0.28^***^ (0.09)	0.31^***^ (0.09)
**Age group** ****×**** **female**												
Young-old × female	16.85 (25.81)	−7.85 (24.08)	17.81 (27.54)	40.91 (26.04)	−27.12 (33.17)	−28.97 (34.65)	41.97 (26.33)	51.23^*^ (25.70)	15.99 (23.14)	14.35 (19.67)	0.09 (0.13)	0.08 (0.13)
Old-old × female	84.94^**^ (31.17)	13.34 (28.05)	29.69 (33.82)	29.93 (32.03)	−18.47 (39.84)	−38.54 (32.45)	43.54 (35.23)	72.10^*^ (33.60)	48.82^*^ (24.16)	27.16 (20.96)	−0.11 (0.16)	−0.17 (0.16)
Oldest-old × female	129.8^*^ (55.19)	43.14 (45.96)	−137.3^*^ (64.8)	−18.50 (59.17)	82.74 (49.87)	−1.93 (29.62)	−124.8 (77.54)	−44.19 (73.16)	80.16^**^ (30.48)	48.84 (28.72)	−0.34 (0.23)	−0.39 (0.22)

* *P < 0.05*,

** *P < 0.01, and*

*** *P < 0.001. This table was based on the analysis of the 2019 (ATUS) respondents aged 55 years or older. All regressions were weighted. Model 1 controlled for race, education, family income, disabilities and functional limitations, and day of time diary. Model 2 added indicators of work and family life, including marital status, living arrangements, and employment status. Coefficients for control variables were omitted for brevity*.

## Discussion

The results of this study documented substantial heterogeneity in daily social contact and time use within the older population. Gaps in daily social contact were found across age groups and by gender. There are three key findings. First, young-old adults and old-old adults showed different daily social contact patterns than mid-life adults. Older adults in these two age groups spent more time with people in the same household but less time in public places, a result that persisted in the fully adjusted models. Second, patterns of daily social contact also differed by gender. Women spent less time alone and visited more public places. Some, but not all, of the gender differences were explained by indicators of work and family life. Third, with age, individuals spent more time alone. However, the age differences in time alone were fully explained by differences in sociodemographic characteristics. These findings have two important implications for vulnerabilities of older adults during the COVID-19 period, namely (1) heterogeneity in risk of infection due to different exposure contexts and (2) psychosocial consequences of social distancing measures.

First, the findings revealed great diversity in the social lives of older adults. In particular, the everyday social contact, time with different groups of people, and time in public places of older adults before the outbreak of the COVID-19 pandemic varied by age group and by gender. As such, should these time use patterns persist during COVID-19, the risk of COVID-19 infection may differ substantially within the older adult population. For example, the young-old and old-old spent more time with family members and less time in public places than mid-life adults, which may make them more vulnerable to COVID-19 infection when close family members are infected. In contrast, older women, who visited more public places, may be more vulnerable to COVID-19 infection from contact with strangers. This heterogeneity in daily social patterns among older adults, if persisting during COVID-19, means the prevailing treatment of older adults as a homogenous group misses important information that is significant for prevention and intervention. The preventive measures recommended by the CDC, such as staying 6 feet away from others and avoiding crowds (26), do not account for the different COVID-19 exposure contexts that older adults have based on their everyday social life and social contact. Results from this study suggest that incorporating information on exposure contexts that differ by age and gender into response strategies can more effectively manage the risk of COVID-19 infection and mitigate its negative impacts. For example, because young-old and old-old adults spend more time with their household members and less time with non-family members and in public places, offering specific steps to manage close interactions at home would better safeguard this population than public social distancing recommendations. Likewise, given that old-old and oldest-old women spend more time in public places, effectively safeguarding this population may mean offering supports for daily activities that help them avoid crowded public places. In this way, public health messaging and response strategies can better protect older adults when it recognizes the heterogeneity in their patterns of social contacts and time use.

Should these heterogeneous patterns of daily social contact and time use persist during COVID-19, the findings of this study also imply that the social distancing measures may not affect all older adults in the same way. For example, when social distancing rules are imposed, older adults who would normally stay longer in public places and visit more public places daily will experience more changes than older adults who would normally spend most of their time with people in the same household. Applying this reasoning alongside the findings of this study suggests that mid-life adults and older women likely experience the most disruption in their daily social lives when social distancing measures are imposed. Importantly, these changes in the daily social lives of mid-life adults and older women may put them at greater risk for poorer mental well-being. Several recent studies suggest that pandemic-induced changes in the personal life of an individual are associated with poorer mental health outcomes (27, 28). However, the existing discourse does not recognize that non-pharmaceutical preventive measures, such as social distancing requirements or stay-at-home orders, will affect subgroups of older adults differently. The findings of this study indicate that mid-life adults and women are likely at higher risk of poor psychosocial consequences when social distancing measures are implemented and, as such, merit the investment of more resources and measures to promote social integration and psychological well-being. For example, mid-life adults and older women would likely benefit from receiving low-cost or free broadband services at home and training on how to use online services (e.g., Zoom software and online grocery order platforms) to meet some of their everyday social and basic needs. In addition, some evidence suggests that frequent telephone contact and video communication from social service organizations can help reduce feelings of social isolation and improve mental health in older adults (29). Based on the findings of this study, interventions of this type would be most effective when targeting at subgroups of older adults who are likely experiencing the most social disruption, such as mid-life adults and women.

In addition to these practical implications, findings from this study add to the growing literature on using the social network perspective to inform prevention and intervention efforts during the pandemic period. In particular, this study extends the focus from social network ties to patterns of daily social contact, complementing prior studies on the social connectedness of older adults while also adding new knowledge to their daily life. For example, although many prior studies suggest that the networks of older adults are kin-centered (30–33), findings show that not all groups of older adults spend more time with immediate family members on a daily basis. In addition, findings also suggest that, within the older adult population, there is a significant difference in time spent alone across age groups and by gender.

This study has several limitations. First, because the ATUS surveys only non-institutionalized individuals, the findings cannot be generalized to an important group to consider the following: older adults in nursing homes, older adults in assisted living facilities, or incarcerated older adult populations. Second, the life of older adults is complex and dynamic (34–36), and the study may not have captured all aspects of that complexity. For example, it is possible that the unprecedented pandemic has not only affected the everyday time use and social contact of older adults but also their social roles. For example, stay-at-home orders may increase conflict in older couples and thus lead to a higher likelihood of marital disruption and, consequently, more individuals living newly alone. A full analysis of the interrelationship between the COVID-19 pandemic and daily social contact, including changing social roles, is beyond the scope of this report because it would require longitudinal data. Data collection that traces the social contacts and time use longitudinally of older adults during the pandemic period would likely shed additional light on the complex social pathways that generate heterogeneity in vulnerabilities among the older population. An additional limitation of this study is that it relies on data from 2019 and, as such, cannot assess the daily social life and social contact of older adults during the pandemic. The release of 2020 data soon will enable direct examination of the pandemic-induced changes in the daily social contact of older adults by comparing patterns from two waves of data. The final limitation of this study is that the ATUS collects data only of the participating respondents instead of all family members. Therefore, this study is not able to incorporate information about the risk of exposure from a spouse, partner, or any other member of the household of an individual.

Limitations notwithstanding, the findings of this study suggest that incorporating heterogeneity in exposure contexts into the understanding of vulnerability may help plan more effective protections for older adults during the COVID-19 pandemic or future pandemics. This study also demonstrates how scholars can use existing data like the ATUS to refine the understanding of infection risk. Since there is currently little detailed information on the everyday social contacts and time use of older adults, governments may feel they are making decisions in the dark. This study demonstrates the potential usefulness of existing social science data to inform real-time practices that better protect a population group, such as older adults.

## Data Availability Statement

Publicly available datasets were analyzed in this study. This data can be found here: https://timeuse.ipums.org/.

## Author Contributions

J-HC designed the study, analyzed the data, and wrote the manuscript.

## Conflict of Interest

The author declares that the research was conducted in the absence of any commercial or financial relationships that could be construed as a potential conflict of interest.

## Publisher's Note

All claims expressed in this article are solely those of the authors and do not necessarily represent those of their affiliated organizations, or those of the publisher, the editors and the reviewers. Any product that may be evaluated in this article, or claim that may be made by its manufacturer, is not guaranteed or endorsed by the publisher.
